# Simultaneous Osteochondral Fractures of the Medial Patella and Lateral Femoral Condyle Following Lateral Patellar Dislocation: A Case Report

**DOI:** 10.7759/cureus.91128

**Published:** 2025-08-27

**Authors:** Akhil Bolisetti, Sreeram Ravi, Robert A Gallo

**Affiliations:** 1 Orthopaedics, Penn State College of Medicine, Hershey, USA; 2 Orthopaedics, Penn State Health Milton S. Hershey Medical Center, Hershey, USA; 3 Orthopaedics and Rehabilitation, Penn State Health Milton S. Hershey Medical Center, Hershey, USA

**Keywords:** lateral femoral condyle, medial patellar facet, mri, musculoskeletal mri and ct, musculoskeletal radiology, muskuloskeletal mri, osteochondral fragment, patellar dislocation, simultaneous osteochondral injuries

## Abstract

Patellar dislocation most commonly occurs laterally and is associated with underlying anatomical abnormalities such as trochlear dysplasia and patella alta. While osteochondral injuries typically involve the lateral femoral condyle, simultaneous osteochondral fractures of the medial patella and lateral femoral condyle are extremely rare and often under-diagnosed due to subtle imaging findings and spontaneous patellar reduction. The purpose of this report is to highlight a rare injury pattern following lateral patellar dislocation and to emphasize the importance of early diagnosis using advanced imaging to prevent long-term complications.

This retrospective case report describes a 22-year-old female patient who presented two weeks after a lateral patellar dislocation with knee pain and limited range of motion. Diagnostic imaging, including CT and MRI, was used to evaluate the extent of injury. The patient underwent surgical intervention consisting of arthroscopic and open fixation of osteochondral fragments using K-wires and ConMed smart nails, along with retinacular release. Postoperative outcomes were monitored over an eight-month period to assess recovery, range of motion, and symptom resolution.

Intraoperatively, a large osteochondral fragment was found embedded in the medial patella, matching a defect in the lateral femoral condyle. Fixation was successful. At one-week follow-up, the patient showed improvement in pain and function. By eight months, she regained full range of motion, ligamentous stability, and resolution of symptoms.

Simultaneous osteochondral injury to the medial patella and lateral femoral condyle is a rare but significant complication of lateral patellar dislocation. Prompt diagnosis and surgical fixation lead to favorable outcomes.

## Introduction

Patellar dislocation occurs in approximately 5.8 per 100,000 adults and is most commonly associated with a pathoanatomical condition that predisposes or increases the risk of patellar dislocation [1]. The most common pathoanatomical conditions that increase the risk of a patient experiencing a dislocation are trochlear dysplasia, patella alta, increased femoral anteversion, increased external tibial rotation, patellar lateral tilt, oblique vastus medialis hypoplasia, varus-valgus deformities, and increased medial patellofemoral ligamentous laxity [1].

Patellar dislocation generally occurs laterally, causing the medial patellar facet to collide with the lateral femoral condyle. As a result, a lateral patellar dislocation will disrupt the medial soft tissue structures and most commonly cause damage to the patellofemoral articular cartilage [2]. MRI studies have shown that approximately 76% of patients experience an articular cartilage injury after a primary patellar dislocation [2]. Additionally, 63% of patients experienced osteochondral fractures (OFC) located on the patella, 34% on the lateral femoral condyle, and only 3% on both the patella and lateral femoral condyle [3]. OFCs were reported to be more frequent in female patients compared to male patients and tended to be larger proceeding a primary dislocation compared to a recurrent dislocation [4]. Simultaneous osteochondral fractures involving the medial facet of the patella and the lateral femoral condyle are rare and have not been commonly reported in adults within orthopedic literature [5]. Only 3% of reported cases have portrayed simultaneous osteochondral fractures [5].

Patients presenting with lateral patellar dislocation are usually unaware that they had a dislocation due to the spontaneous reduction of the patellar that transiently occurs proceeding the dislocation [5]. This has contributed to the misdiagnosis pre-operatively as a meniscal or ligamentous injury [5]. Additionally, OFCs of the patella most commonly follow a patellar dislocation but are often missed on primary radiographs taken after trauma due to the nature of these fractures often being difficult to interpret on radiographs and requiring multiple projections and a strong clinical suspicion to make a proper diagnosis [5]. In a study conducted by Stanitski et al., only 11 out of 48 radiographs could be used to diagnose an osteochondral defect [6]. Therefore, the rarity of the simultaneous lateral femoral condyle and medial patellar osteochondral fractures combined with difficulty in interpreting radiographs and CT scans can lead to potentially increased pre-operative misdiagnosis.

## Case presentation

This patient is a 22-year-old female patient with no significant past medical history who presented for a left patellar subluxation event that she had experienced about two and a half weeks prior. She was kneeling on an airplane and noticed sudden pain in the medial aspect of her left knee after her knee fell into a divot in the floor. She had knee pain for a few days after the incident, but there was no swelling. About five days after the initial event, she felt her patella dislocate laterally after standing up.

**Figure 1:**
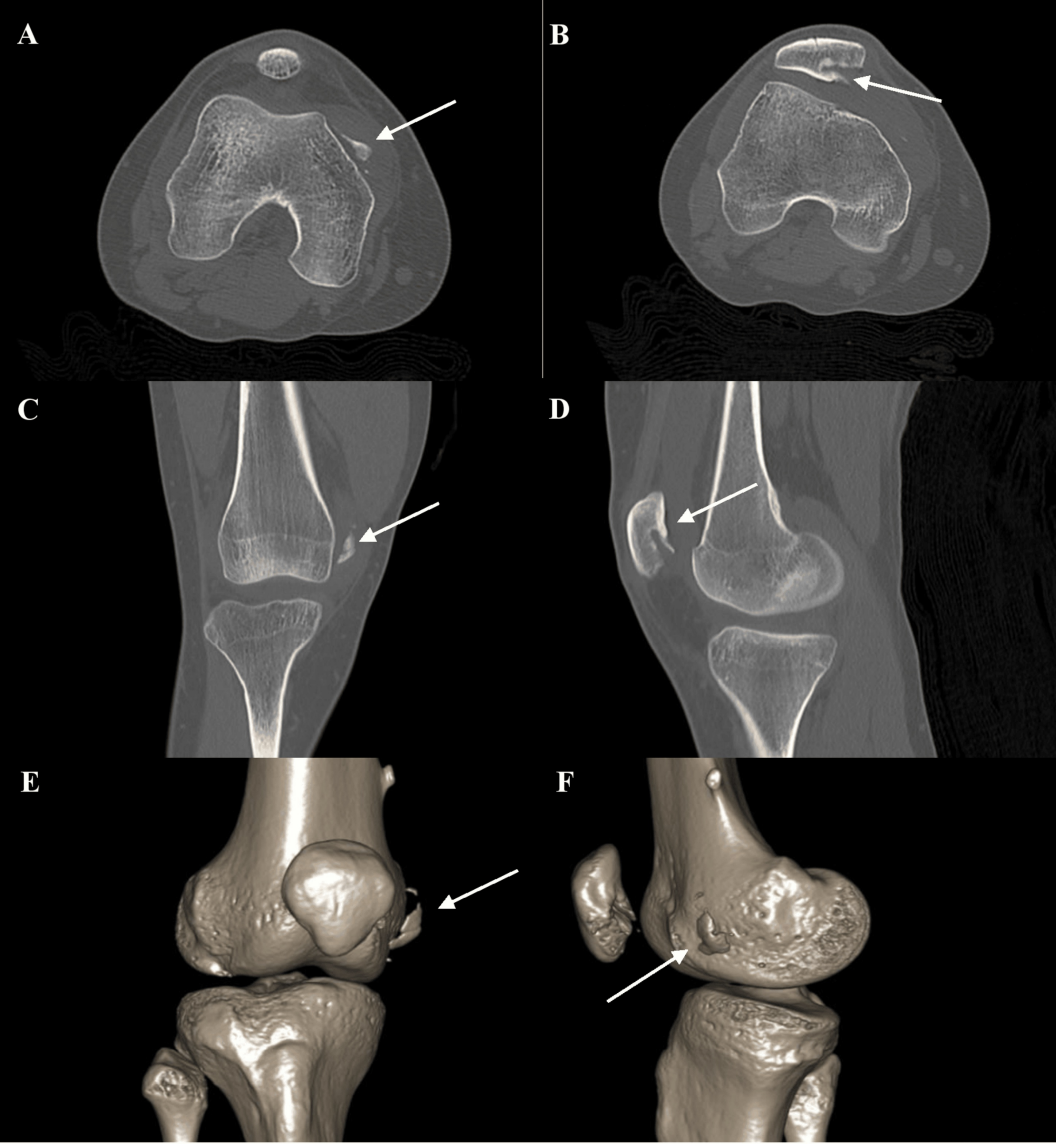
Pre-operative axial (A, B), sagittal (C, D), and CT-based three-dimensional reconstructions (E, F) suggestive of a large osteochondral fragment in the medial gutter consistent in shape with a defect of the inferior aspect of the medial patellar facet (A, C, E, F), with an osteochondral fragment of the posterior lateral femoral condyle embedded inside the inferior aspect of the medial patellar facet (B, D). A: Pre-operative axial CT image suggestive of large osteochondral fragment in medial gutter consistent in shape to defect of inferior aspect of medial patellar facet. B: Pre-operative axial CT image suggestive of osteochondral fragment of posterior lateral femoral condyle embedded inside of the inferior aspect of the medial patellar facet. C: Pre-operative sagittal CT image suggestive of large osteochondral fragment in medial gutter consistent in shape to defect of inferior aspect of medial patellar facet. D: Pre-operative sagittal CT image suggestive of osteochondral fragment of posterior lateral femoral condyle embedded inside of the inferior aspect of the medial patellar facet. E: Pre-operative CT based three-dimensional bone reconstruction of lateral gutter suggestive of large osteochondral fragment in medial gutter consistent in shape to defect of inferior aspect of medial patellar facet. F: Pre-operative CT based three-dimensional bone reconstruction of medial gutter suggestive of large osteochondral fragment in medial gutter consistent in shape to defect of inferior aspect of medial patellar facet. White arrows in A,C,E,F osteochondral fragment consistent in shape to defect of inferior aspect of medial patellar facet and those in B and D osteochondral fragment of posterior lateral femoral condyle embedded inside of the inferior aspect of the medial patellar facet. Images reconstructed at Penn State 3D Reconstruction Lab.

She presented to an outside facility where radiographs and CT scan showed a patellar dislocation with free patellar fragments (Figure 1). One week later, an MRI of her left knee showed a posterior cruciate ligament tear. She remained weightbearing as tolerated and had no similar injuries prior. She presented to our outpatient clinic for surgical evaluation.

On presentation, her right knee had significant effusion, most prominent in the suprapatellar and infrapatellar regions. The patella was mobile without any mechanical locking or catching upon passive manipulation. The patient was tender about the medial joint line. Range of motion was limited from 0-80 degrees. She had negative anterior drawer, posterior drawer test, and Lachman's test bilaterally. Her contralateral patella was examined and demonstrated hypermobility at baseline.

**Figure 2:**
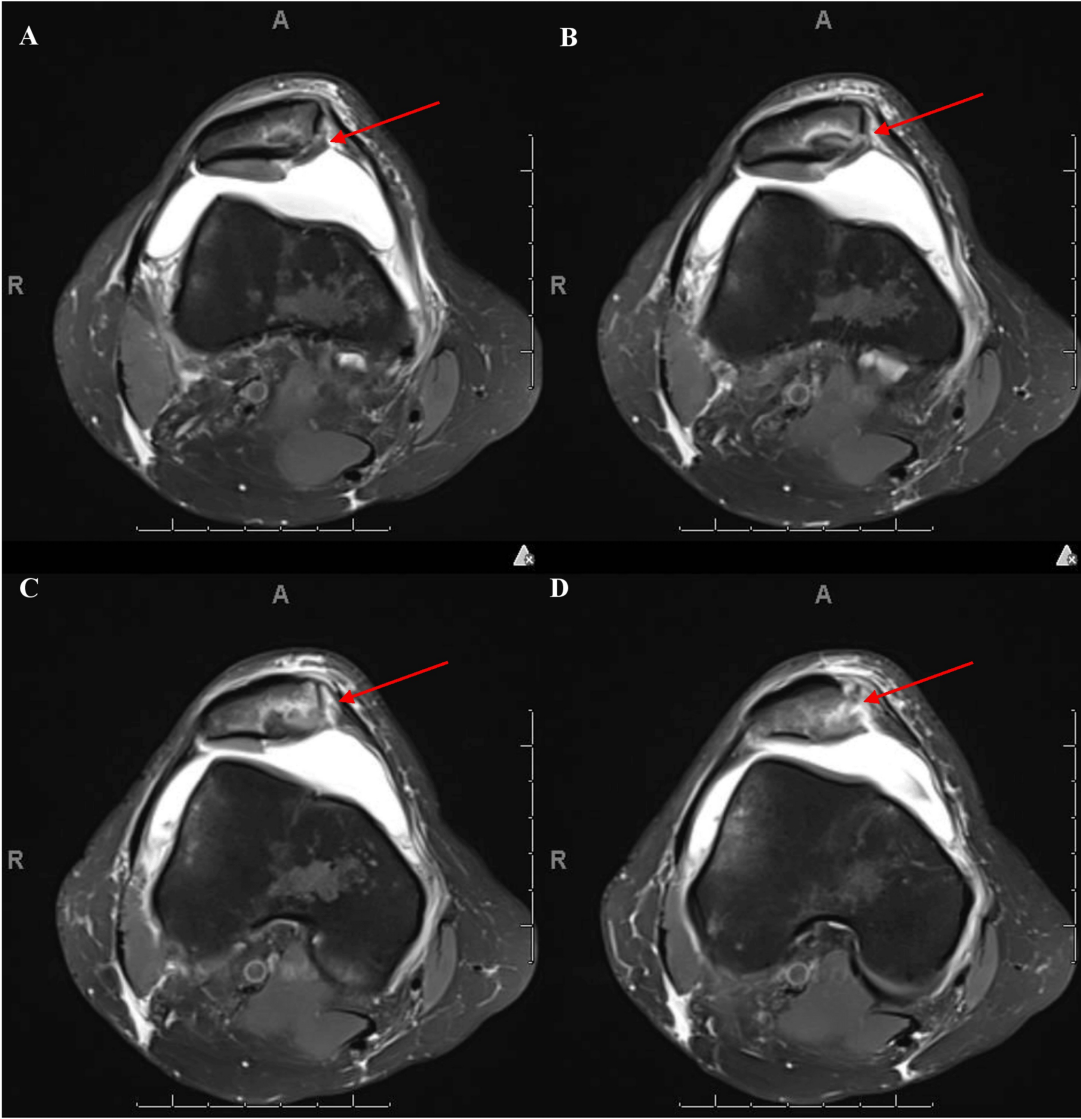
Pre-operative axial MRI images (A, B, C, D) demonstrate an osteochondral fragment of the posterior lateral femoral condyle embedded within the medial patellar facet - proximal inferior aspect with associated elevation of patella (A, B), and distal inferior aspect with (C) and without (D) evidence of healing. A: Pre-operative axial MRI image suggestive of osteochondral fragment of posterior lateral femoral condyle embedded inside of the proximal inferior aspect of the medial patellar facet and causing elevation. B: Pre-operative axial MRI demonstrating an osteochondral fragment from the posterior lateral femoral condyle, embedded within the middle inferior aspect of the medial patellar facet, resulting in elevation. C: Pre-operative axial MRI image suggestive of osteochondral fragment of posterior lateral femoral condyle embedded inside of the distal inferior aspect of the medial patellar facet with evidence of healing. D: Pre-operative axial MRI image suggestive of osteochondral fragment of posterior lateral femoral condyle embedded inside of the distal inferior aspect of the medial patellar facet. Red arrows in all the figures indicate the osteochondral fragment of posterior lateral femoral condyle embedded inside of the inferior aspect of the medial patellar facet.

Additional imaging findings demonstrated a subacute right lateral femoral condyle fracture with the presence of a large bone fragment in the medial aspect of the knee as well as a chondral flap on the patellofemoral surface, suggestive of subacute subchondral fracture (Figure 2).

Ultimately, a diagnosis of right knee patellar dislocation with osteochondral fracture was made. She was scheduled for right knee arthroscopy with probable open reduction and internal fixation of osteochondral fracture with possible medial patellofemoral ligament reconstruction. The knee was visualized using medial and lateral arthroscopic portals. Due to evidence of large grade IV chondromalacia at the posterior aspect of the lateral femoral condyle and medial inferior patellar facet, a decision was made to convert the procedure to an open incision. The medial portal was extended 5 cm distally and proximally into a medial parapatellar approach. 

**Figure 3:**
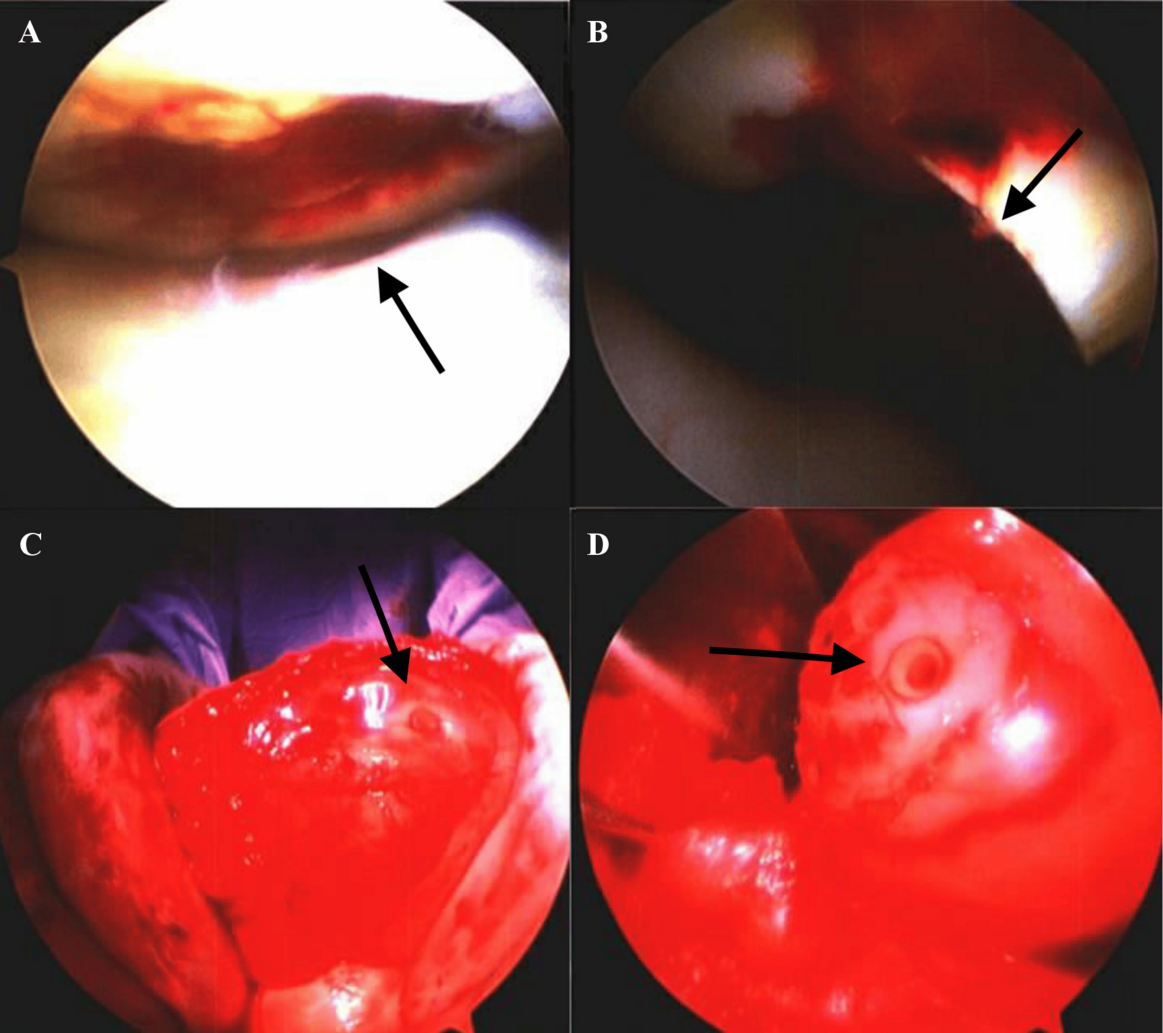
Intra-operative images show an osteochondral defect of the posterior lateral femoral condyle embedded in the inferior aspect of the medial patellar facet visualized arthroscopically from the medial (A) and posterior (B) aspects of the right patella. Open approach demonstrating removal of the defect and re-insertion into patella of a large loose osteochondral fragment found in the medial gutter (C, D). A: Intra-operative arthroscopic images of medial aspect of right patella portraying osteochondral defect of posterior lateral femoral condyle embedded inside inferior aspect of medial patellar facet. B: Intra-operative arthroscopic images of posterior aspect of right patella portraying osteochondral defect of posterior lateral femoral condyle embedded inside inferior aspect of medial patellar facet. C: Intra-operative open image of right posterior patellar surface portraying removal of osteochondral defect of posterior lateral femoral condyle and re-insertion of large loose patellar osteochondral fragment found in medial gutter. D: Intra-operative open image of right posterior patellar distal surface portraying removal of osteochondral defect of posterior lateral femoral condyle and re-insertion of large loose patellar osteochondral fragment found in medial gutter. Black arrows in A,B indicate osteochondral fragment of posterior lateral femoral condyle embedded inside of the inferior aspect of the medial patellar facet and those in C, D the removal of osteochondral fragment of posterior lateral femoral condyle embedded inside of the inferior aspect of the medial patellar facet.

The medial facet defect was found to have an osteochondral defect embedded inside of the inferior aspect of it. This caused the subchondral bone of the medial patellar facet to be elevated. A large loose osteochondral fragment was encountered in the medial gutter, which was consistent in shape with the defect about the medial patellar facet. The fragment was emancipated from the surrounding subchondral bone, prepared and inserted into the osteochondral defect about the posterior lateral femoral condyle utilizing a K-wire and four ConMed SmartNails (proprietary 96L/4D polylactic acid (PLA) 96% L monomer/4%D monomer) polylactide; ConMed, Utica, NY, USA). The larger fragment that was found in the medial gutter was fixated to the medial patellar facet (Figure 3). Following arthrotomy, a retinacular release was performed to improve the patient’s patellar tracking. The joint was irrigated and the arthrotomy incision was closed.

**Figure 4:**
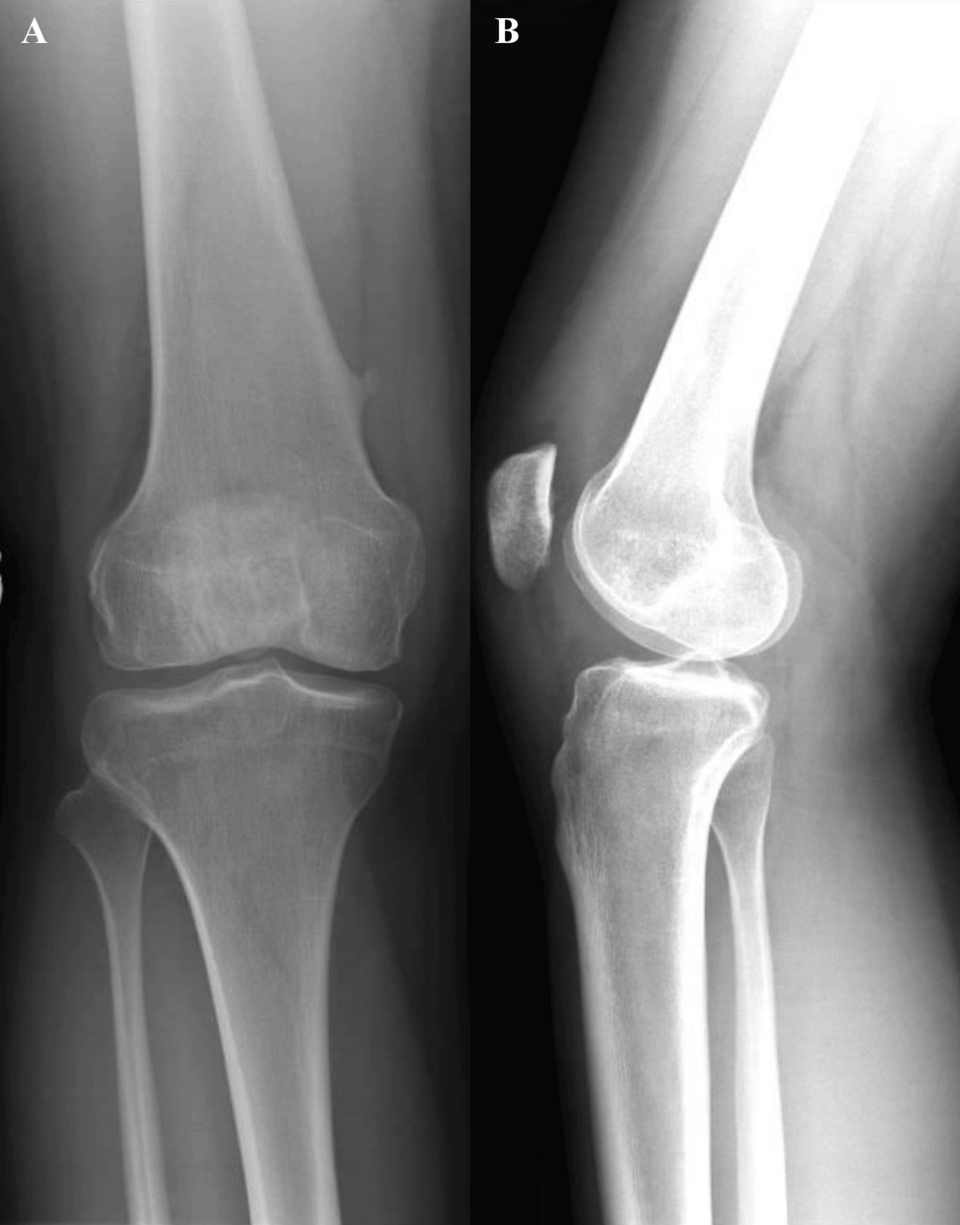
Eight-month post-operative X-rays demonstrate removal of the osteochondral defect from the inferior aspect of the medial patellar facet, with coronal view (A) showing re-insertion into the posterior lateral femoral condyle and sagittal view (B) depicting the patellofemoral surface post-removal. A: Post-operative coronal X-ray of posterior tibiofemoral joint portraying removal of osteochondral defect from inferior aspect of medial patellar facet and re-insertion into posterior lateral femoral condyle. B: Post-operative sagittal X-ray of patellofemoral surface portraying removal of osteochondral defect from inferior aspect of medial patellar facet.

**Figure 5:**
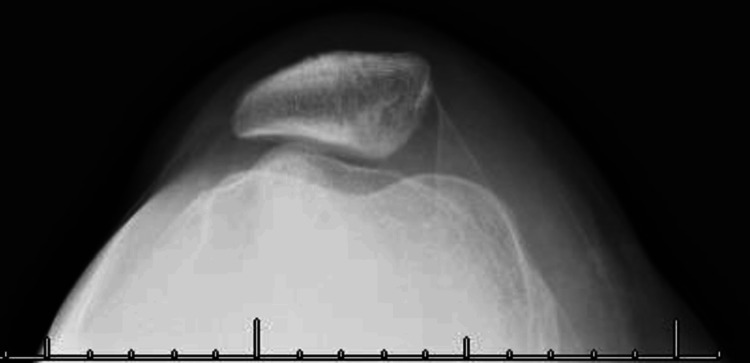
Post-operative axial X-ray of patellofemoral articulation portraying removal of osteochondral defect from inferior aspect of medial patellar facet.

Postoperatively, the patient had no weightbearing restrictions and was placed in a hinged knee brace locked in extension. Her initial follow-up at one week demonstrated improvement in clinical outcome with reduced pain and no deficits with ambulation. She continued therapy physically from one week to eight months postoperatively. At eight-month follow-up, she had attained full range of motion and ligamentous stability, with no pain or effusion (Figures 4, 5).

## Discussion

In this case, we demonstrate a unique clinical presentation of an osteochondral fragment from the lateral femoral condyle embedded within the patella. Patellar dislocation generally occurs laterally, causing the medial patellar facet to collide with the lateral femoral condyle. The patient was unique because of the presence of an osteochondral fragment from the lateral femoral condyle that was embedded within the patella, adhered to the subchondral bone of the medial facet, and had already begun healing. The smaller and larger osteochondral fragments were pulled out and keyed back into the lateral femoral condyle and the medial patellar facet, respectively. 

Previous studies have indicated proceeding with osteochondral fragment removal and medial patellofemoral ligament (MPFL) reconstruction, with a Gracilis graft, for an acute primary patellar dislocation with the presence of a loose body [3,7]. This method was effective in stabilization, reducing the rate of instability post-operation, improved return to sports, and required fewer subsequent stabilization surgeries compared to providing no treatment or an MPFL repair [7]. The study conducted by Aydoğmuş et al. indicated an alternative method by fixating the fractured fragment back into its original position using selected headless cannulated compressions screws as it was thought to achieve greater stability [8]. The desired positive results of fixation and osteosynthesis were attained, and normally there is no requirement for a second surgical procedure to remove the headless cannulated compressions screws [8]. However, four months post-operatively, although full range of motion was attained, the patient within the study was experiencing friction in the patellar-femoral joint that led to cartilage destruction, pain, restriction, and the eventual removal of the headless screws. Two months following the removal of screws, the patient returned to full range of motion and all complaints regarding friction, pain, and restriction were resolved [8]. Aydoğmuş et al. further highlighted the importance of embedding screws deeply below cartilage surface to avoid post-operative complications, or to use absorbable and adhesive alternative fixation methods [8]. In this case, K-wire and four ConMed smart nails were utilized to fixate the smaller osteochondral fragment to the posterior lateral femoral condyle and the larger fragment to the medial patellar facet. At one week post-operatively, the patient made good recovery, and her pain was well controlled.

Ultimately, the risk of misdiagnosis can lead to the patient being untreated and would increase the risk for post-traumatic arthritis in younger individuals [2,5]. Patients who had experienced a lateral patellar dislocation were significantly more likely to develop and be diagnosed with arthritis compared to patients that did not experience a patellar dislocation [2,9]. Numerous factors have been identified and are predictive of arthritis post patellar dislocation including the presence of osteochondral injury and OFCs [2]. Additional factors include recurrent instability, injury to the articular cartilage and subchondral bone, and trochlear dysplasia [2]. Injury to the articular cartilage and subchondral bone led to accelerative degenerative changes due to increased joint inflammation that altered the distribution of stress across the patellofemoral joint [2]. Trochlear dysplasia also likely accelerated degeneration due to significantly altering the contact pressure, leading to recurrent stress being applied to the patellofemoral joint [2]. Currently, according to the imaging algorithm, plain radiographs are first used in diagnosis, but MRI has shown to have a higher rate of OFC detection compared to plain radiograph alone and should be considered routinely in patients with primary patellar dislocation [10]. Therefore, the most important factor in preventing the development of both degenerative arthritis and the osteochondral fragments is early diagnosis and management of the lateral patellar dislocation.

## Conclusions

This case highlights a rare presentation of simultaneous osteochondral fractures of the medial patella and lateral femoral condyle following a lateral patellar dislocation. This injury pattern is often underrecognized due to subtle radiologic findings and spontaneous patellar reduction. Prompt recognition using advanced imaging modalities such as MRI, followed by appropriate surgical management, can result in improved functional recovery and help prevent long-term sequelae such as chronic instability and post-traumatic osteoarthritis. Clinicians should maintain a high index of suspicion for complex osteochondral injuries in the setting of patellar dislocation, particularly when clinical symptoms are disproportionate to initial radiographic findings. This case reinforces the importance of thorough evaluation and timely intervention to optimize outcomes in young, active patients.
